# Draft genome sequence of *Zygosaccharomyces siamensis* kiy1 isolated from unpasteurized honey

**DOI:** 10.1128/mra.00725-24

**Published:** 2024-11-27

**Authors:** Kan Iwata, Daisuke Minegishi, Mayumi Maeda, Kenji Maehashi, Jun Yoshikawa

**Affiliations:** 1 Department of Fermentation Science and Technology, Graduate School of Applied Bioscience, Tokyo University of Agriculture, Setagaya-ku, Tokyo, Japan; 2 Department of Contract Analysis and Software Development Services Group, Tohoku Chemical Co., Ltd., Morioka-shi, Iwate, Japan; 3 Department of Fermentation Science, Faculty of Applied Bioscience, Tokyo University of Agriculture, Setagaya-ku, Tokyo, Japan; University of California Riverside, Riverside, California, USA

**Keywords:** *Zygosaccharomyces siamensis*, arabitol, honey, sugar alcohol

## Abstract

In this study, a draft genome sequence of *Zygosaccharomyces siamensis* kiy1 isolated from unpasteurized honey in Japan was analyzed. This strain was reported to be a promising arabitol-producing strain, and this report contributes to a better understanding of arabitol production in yeasts.

## ANNOUNCEMENT

*Zygosaccharomyces siamensis*, a sugar-tolerant yeast, was isolated in Thailand as a new species in 2011 (1). Recently, arabitol production by *Z. siamensis* kiy1 was reported (2). Arabitol, a five-carbon sugar alcohol, is attracting attention as an alternative sweetener because of its low calorie content, low glycemic index, and ability to improve insulin resistance (3). Strain kiy1 was isolated from unpasteurized *Apis mellifera* honey collected in Japan using a high-concentration glycerol medium (2). The internal transcribed spacer (ITS) region was amplified by PCR using KOD FX (TOYOBO, Osaka, Japan) with ITS1 and ITS4 primers. DNA sequences were analyzed using the BLAST version 2.13.0 (https://blast.ncbi.nlm.nih.gov/Blast.cgi) against sequences registered in the DNA Data Bank of Japan (DDBJ)/European Nucleotide Archive (ENA)/GenBank database. The obtained sequence (GenBank accession number: LC727651) was aligned with the ITS sequences of other *Zygosaccharomyces* spp. type strains using MEGA 11 version 11.0.13, and a molecular phylogenetic tree was constructed with the neighbor-joining method. The sequence showed 99.32% identity to *Z. siamensis* JCM 16825^T^ by BLAST analysis, and strain kiy1 was identified as *Z. siamensis* by molecular phylogenetic tree analysis.

The colony of *Z. siamensis* kiy1 was suspended in 10 mM Tris-HCl buffer (pH 8.0) containing 1 mM ethylenediaminetetraacetic acid, and the cell pellet was collected by centrifugation at 15,000 × *g* for 5 min. Genomic DNA was prepared using a Dr. GenTLE (for yeast) High Recovery Kit (Takara Bio Inc., Shiga, Japan). Approximately 1.5 µg of DNA was subjected to whole-genome sequencing. The amount of DNA was determined by absorbance using a BioSpectrometer kinetic (Eppendorf, Hamburg, Germany). The genome sequence of *Z. siamensis* kiy1 was performed at Eurofins Genomics K.K. (Tokyo, Japan). DNA libraries were prepared using TruSeq DNA PCR-Free (Illumina, San Diego, CA, USA) according to the manufacturer’s protocol. The prepared library was sequenced at 2  ×  151 bp using a HiSeq X (Illumina). Adapter sequences, sequences of less than 20 base reads, and other unwanted sequences were removed from the sequenced paired-end reads using Cutadapt version 4.6 (4). Trimmed and filtered reads were assembled using SPAdes version 3.15.5 with the isolate and k options changed from the default settings (-k 21, 33, 55, 77) to (-k 31, 51, 71, 91, 111) (5).

The number of reads were 127,128,528 , and the sequenced base pairs were 19,237 Mbp. The GC content of sequenced bases was 38.85%, and the Q30 score was 93.9%. The *de novo* assembled genome sequence calculated using QUAST version 5.2.0 (6) was 9,655,933 bp. The statistics of the genome from *Z. siamensis* were summarized in Table 1. The genome sizes of the already registered *Z. siamensis* strains MinabeTanabe (GCA_013423405.1) and CBS 12273 (GCA_030574045.1) were not significantly different from the kiy1 strain (Table 1). The genome assembly quality was assessed using BUSCO version 5.5.0 (7), and the completed BUSCO value for the saccharomycetes_odb10 data set was 99.8% (2,132 out of 2,137 genes). The BUSCO value of 99.8% showed the high reliability of this assembly. Here, we report the draft genome sequence of *Z. siamensis* kiy1, which is considered a promising arabitol-producing strain and contributes to a better understanding of arabitol production by yeast.

**TABLE 1:** Statistics of the genome sequences of *Zygosaccharomyces siamensis*

Statistics	Strains		
	kiy1	MinabeTanabe	CBS 12273
WGS project	BAABWA01	BLZB01	JAJMEI01
Genome size (Mb)	9.7	9.7	9.6
Number of contigs	472	293	283
N50 (kb)	555.5	342.5	122.4
L50	7	9	25
GC(%)	39	39	39
Genome coverage	60.0	60.0	26.7

## Data Availability

The whole-genome shotgun project is available from the DNA Data Bank of Japan (DDBJ, Shizuoka, Japan) repository. DDBJ/ENA/GenBank accession numbers for *de novo* genome sequencing: BAABWA010000001-BAABWA010000467, raw reads accession number: DRR582747, BioProject accession number: PRJDB17565, and BioSample accession number: SAMD00747573.
